# A Subset of Caveolin-1 Interacts with a Fraction of Acyl-CoA:Cholesterol Acyltransferase 1 (ACAT1/SOAT1) at an Endoplasmic Reticulum Subdomain to Attenuate Cholesteryl Ester Biosynthesis

**DOI:** 10.3390/biom16060838

**Published:** 2026-06-08

**Authors:** Catherine C. Y. Chang, Toyoshi Fujimoto, Yoshio Yamauchi, Yasuomi Urano, Ta Yuan Chang

**Affiliations:** 1 Department of Biochemistry and Cell Biology, Geisel School of Medicine at Dartmouth, Hanover, NH 03755, USA; 2 Graduate School of Medicine, Juntendo University, Tokyo 113-8421, Japan; t.fujimoto.xl@juntendo.ac.jp; 3 Graduate School of Agricultural and Life Sciences, The University of Tokyo, Tokyo 113-8657, Japan; yoshio-yamauchi@g.ecc.u-tokyo.ac.jp; 4 Graduate School of Life and Medical Sciences, Doshisha University, Kyoto 610-0394, Japan; yurano@mail.doshisha.ac.jp

**Keywords:** caveolin-1, caveolae, acyl-CoA:cholesterol acyltransferase, sterol O-acyltransferase, cholesterol, cholesteryl oleate, mitochondria-associated membranes, endoplasmic reticulum, trans-Golgi network

## Abstract

Caveolin-1 is a scaffolding protein of caveolae, flask-shaped membrane microdomains involved in diverse cellular processes. Caveolae are primarily localized to the plasma membrane, the trans-Golgi network, and mitochondria-associated endoplasmic reticulum (ER) membranes (MAMs). Most enzymes involved in cholesterol biosynthesis reside in the ER, and although caveolin-1 avidly binds cholesterol, its role in cholesterol trafficking remains unclear. Acyl-coenzyme A:cholesterol acyltransferases (ACAT1 and ACAT2) convert free cholesterol into cholesteryl esters for storage, with ACAT1 serving as the predominant isoenzyme in most cell types. ACAT1 is an ER-resident protein, with a fraction associated with specialized ER subdomains, including the MAM. Here, we report that a subset of caveolin-1 molecules appears to be associated with a fraction of ACAT1 in ER subdomains. Using immunoprecipitation under detergent conditions, immunoadsorption of MAM-enriched membranes under detergent-free conditions, and electron microscopy, we provide evidence consistent with an association between a subset of caveolin-1 molecules and ACAT1. Functionally, in mouse embryonic fibroblasts, we show that genetic ablation of caveolin-1 significantly increases the esterification of low-density lipoprotein-derived cholesterol, suggesting that caveolin-1 may attenuate ACAT1 activity. Collectively, these findings indicate that caveolin-1 may modulate cholesterol esterification and contribute to the regulation of cholesterol distribution among cellular membranes.

## 1. Introduction

Caveolins (Cavs) are scaffolding proteins found in caveolae, which are flask-shaped, lipid raft invaginations of the plasma membrane (PM) and selected internal membranes. Cavs are involved in numerous cellular functions, including lipid homeostasis, metabolism, membrane transport, and cell signaling [1,2,3,4]. Caveolins are expressed in a wide range of tissues and bind cholesterol with high affinity [5], as well as sphingolipids such as GM1 [6].

In mouse embryonic fibroblasts (MEFs), Cavs and PM caveolae participate in cellular cholesterol efflux and cholesterol recycling, as reviewed by Fielding and Fielding [7]. In human fibroblasts, *Cav1* mRNA expression is upregulated by free cholesterol and downregulated by oxysterols [8]. These findings indicate that Cav1 and caveolae play important roles in the regulation of cholesterol homeostasis. Lisanti and colleagues demonstrated that Cav1 deficiency in mouse embryonic fibroblasts leads to an increase in cellular cholesteryl ester (CE) content, accompanied by a depletion of free cholesterol relative to wild-type cells [9]. At the molecular level, Cavs were shown to directly interact with ABCA1 [10], the major cellular cholesterol efflux protein [11]; however, direct interactions between Cavs and other key proteins or enzymes involved in cholesterol metabolism had not been clearly established [12].

In addition to the PM, Cavs localize to multiple intracellular compartments, including the trans-Golgi network (TGN) [13,14], mitochondria [15,16,17], extracellular vesicles such as exosomes [16,17], the endoplasmic reticulum (ER) [18,19,20], and ER–mitochondria-associated membranes (MAMs) [21]. The MAM fraction is particularly enriched in cholesterol and ceramides, which are key lipid components that form lipid rafts [22,23]. Collectively, these studies support a model in which Cav1 and caveolae facilitate the exchange and transport of selected lipid components—especially cholesterol—across multiple cellular membranes.

The low-density lipoprotein (LDL) receptor–mediated pathway controls cholesterol homeostasis in many cell types [24,25]. LDL carries cholesteryl linoleate as its major lipid cargo. LDL present in the culture medium binds to its cognate receptor at the PM and undergoes clathrin-mediated endocytosis, entering the endosomal/lysosomal compartments. Within the acidic lysosome, cholesteryl linoleate is hydrolyzed to free cholesterol and free linoleate by lysosomal acid lipase. The liberated cholesterol then exits the endo/lysosomal compartment and traffics to the ER. A portion of this cholesterol is re-esterified by acyl-CoA:cholesterol acyltransferases (ACATs; also termed sterol O-acyltransferases, SOATs) in the ER to form cholesteryl esters, which are stored in lipid droplets, while the remaining cholesterol is distributed to various cellular membranes for recycling via largely unknown mechanisms, as reviewed in [26].

Mammalian cells express two ACAT isoforms, ACAT1 and ACAT2 (also termed SOAT1 and SOAT2). Both isoforms primarily reside in the ER and are allosteric enzymes regulated by cholesterol and oxysterols [26,27]. In the present study, we report the novel finding that portions of ACAT1 and ACAT2 form complexes with Cavs in specific membrane compartments. We further describe the consequences of Cav1 deficiency on the esterification of LDL-derived cholesterol in mouse embryonic fibroblasts and provide a hypothesis on how Cavs might participate in regulating intracellular cholesterol esterification.

## 2. Materials and Methods

### 2.1. Materials

Fetal bovine serum was obtained from Sigma (St. Louis, MO, USA). OptiPrep was purchased from STEMCELL. [^3^H]-labeled acetate and [^3^H]-labeled oleate were purchased from PerkinElmer (Waltham, MA, USA). All other chemicals, of analytical grade, were obtained from Sigma-Aldrich or Fisher Scientific (Waltham, MA, USA). Low-density lipoproteins (LDL) were isolated from fresh human serum, and delipidated serum (~98% depletion of total cholesterol content) was prepared from fetal bovine serum stocks.

### 2.2. Animal Use and Maintenance

Mice were fed ad libitum with a standard chow diet and maintained in a pathogen-free facility in individually ventilated cages under a 12 h light/dark cycle. All animal procedures were approved by the Dartmouth Animal Research Center Institutional Animal Care and Use Committee (IACUC; protocol no. 00002125 (m2)). Animals were monitored daily throughout their lifespan.

### 2.3. Mouse Strains

The *Acat1*^−/−^ (*Soat1* knockout, KO) mouse, a global ACAT1-deficient line originally generated by Meiner et al. [28], was obtained from Dr. Sergio Fazio and maintained on a C57BL/6 background. The *Soat1* KO mouse on a C57BL/6 background is now commercially available from The Jackson Laboratory (Bar Harbor, ME, USA). Caveolin-1 knockout mice (*Cav1* KO; B6.Cg-*Cav1*^tm1Mls^/J) were purchased from The Jackson Laboratory.

### 2.4. Antibodies

Primary antibodies used in this study are listed below, including their source and identifier (Table 1):

### 2.5. Cell Fractionation to Prepare Detergent-Resistant and Detergent-Sensitive Membranes

Cells grown in 100 mm culture dishes were treated with 1.0 mL of TNE buffer (25 mM Tris-HCl, pH 7.5; 150 mM NaCl; 5 mM EDTA) containing a protease inhibitor cocktail from Sigma as previously described [29]. Cells were extracted with 1.0% Triton X-100 (Sigma_Aldrich Corp, St. Louis, MO, USA) for 30 min at 4 °C or with 1.0% Brij-98 (Thermo Scientific Chemicals, Waltham, MA, USA) for 10 min at 37 °C.

Following detergent treatment, cell lysates were harvested and homogenized with 10 strokes using a Dura-Grind stainless-steel Dounce tissue grinder (Wheaton: 1501 N 10th St., Miliville, NJ, USA). Homogenates were centrifuged twice at 1000× *g* for 5 min to remove nuclei and debris. The resulting post-nuclear supernatant fractions were centrifuged at 100,000× *g* for 60 min to isolate detergent-resistant membranes (DRMs) as the pellet and detergent-sensitive membranes (DSMs) as the supernatant. When CHAPSO was used as the detergent, a procedure similar to that used with Triton X-100 was employed.

### 2.6. Immunodepletion and ACAT Activity Assays

ACAT1 is a membrane protein but can be completely solubilized while retaining enzymatic activity with the detergent CHAPS and KCl, as shown in [30]. Immunodepletion and ACAT activity assays were performed as previously described [27]. Briefly, 1.2 × 10^7^ human fibroblasts or CHO cells were seeded in 150 cm^2^ culture dishes and solubilized in buffer containing 2.5% CHAPS and 1 M KCl, while maintaining the total protein concentration at approximately 2 mg/mL. Cell extracts were centrifuged at 100,000× *g* for 60 min to obtain the supernatant; the supernatants were used as the starting material for immunodepletion. The immunodepletion experiments were conducted using specific antibodies against ACAT1 or ACAT2. Non-specific antibodies, added in equal amounts of protein, were used as negative controls. Specific anti-ACAT antibodies form complexes with ACAT; these complexes are pelleted using a microfuge at 14,000× *g* for 1 min, while non-specific antibodies fail to do so [27]. After immunodepletion, ACAT activities in the supernatants and pellets were monitored using a reconstituted assay in liposomes, as described in [27]. Similar experiments were conducted for ACAT2.

### 2.7. Cell Culture

CHO cells, human skin fibroblasts (Hf), HeLa cells, and mouse embryonic fibroblast (MEF) cells were routinely maintained at 37 °C in a 5% CO_2_ incubator as monolayers in either F-12/DMEM (1:1) medium (for CHO cells), DMEM (for Hf and MEF cells), or MEM (for HeLa cells), supplemented with 10% fetal bovine serum (FBS), MEM non-essential amino acids (Gibco, Waltham, MA, USA), and penicillin/streptomycin (Corning, New York, NY, USA). For the experiments shown in Figure 6, cells were cultured in medium supplemented with 5% delipidated fetal serum and 35 µM oleic acid. Cells were detached by treatment with 0.25% trypsin solution (Corning) at 37 °C. Stable transfectants expressing either His-ACAT1 or His-ACAT2 were isolated and described previously [29]. Hf cells and HeLa cells were obtained from ATCC. Mouse embryonic fibroblasts (MEFs) were isolated as previously described [31]. Experiments were performed using cells grown in duplicate or triplicate dishes.

### 2.8. OptiPrep Subcellular Fractionation and Western Blot Analysis

OptiPrep density gradient centrifugation and Western blot analyses were performed as previously described [32,33]. Cells grown to near confluence in 150 mm dishes were washed twice with phosphate-buffered saline (PBS) and once with homogenization buffer (250 mM sucrose, 20 mM Tris-HCl, pH 7.4, 1 mM EDTA). Cells were harvested in 1 mL of homogenization buffer containing protease inhibitors and homogenized using a stainless-steel homogenizer with 40 strokes.

Post-nuclear supernatants were layered onto an 11 mL discontinuous 5–25% OptiPrep gradient and centrifuged at 200,000× *g* (40,000 rpm) for 3 h in a Beckman SW41 rotor. Fourteen fractions (800 µL each) were collected from the top and analyzed by Western blotting. Band intensities were normalized to vinculin or β-tubulin and quantified using Image J, Version 2.1.0/1.53c (National Institutes of Health (NIH), Bethesda, MD, USA).

### 2.9. Immunoadsorption of ER-Enriched Fractions

Following OptiPrep density gradient centrifugation of His-ACAT1–expressing CHO cells [30] or HeLa cells, equal amounts of fractions 9–11 (from CHO) or fraction 10 (from HeLa) were incubated with anti-ACAT1 IgG or rabbit IgG (negative control) for 1 h, followed by incubation with µMACS Protein A beads for 30 min. After extensive washing, bound proteins were eluted with 1× SDS–PAGE sample buffer. Input, flow-through, and eluate fractions were analyzed by immunoblotting using the indicated antibodies in a similar manner as described in [33].

### 2.10. High-Resolution Electron Microscopy

ER-enriched fractions (fractions 9–11) obtained from OptiPrep fractionation were pooled and ultracentrifuged at 100,000× *g* for 45 min at 4 °C to collect the pellets. Pellets were fixed with 1% paraformaldehyde for 15 min, diluted with PBS to a final paraformaldehyde concentration of 0.1%, and ultracentrifuged again. Final pellets were resuspended in PBS and processed for electron microscopy. High-resolution freeze-fracture replica labeling was performed as previously described [34] using anti-His IgG followed by immunogold-labeled secondary antibodies.

### 2.11. LDL-Derived Cholesterol Trafficking Assay

The LDL-derived cholesterol trafficking assay described by Goldstein et al. [25] and modified by Sugii et al. [32] and Cadigan et al. [35] was used to monitor percentage uptake, hydrolysis and re-esterification of LDL-associated [^3^H]-cholesteryl linoleate. Percent [^3^H]-CL-LDL uptake is defined as the amount of [^3^H]-CL-LDL remaining in the cell extracts divided by the total counts of [^3^H]-CL-LDL added to cells during the pulse. Hydrolysis was calculated as the sum of [^3^H]-cholesterol and [^3^H]-cholesteryl oleate divided by the amount of [^3^H]-cholesteryl linoleate taken up by the cells. Re-esterification was calculated from the amount of [^3^H]-cholesteryl oleate formed divided by the sum of [^3^H]-cholesterol and [^3^H]-cholesteryl oleate.

### 2.12. Statistical Analysis

Statistical analyses were performed using a two-tailed, unpaired Student’s *t*-test with GraphPad Prism 10 (GraphPad Software, San Diego, CA, USA). Differences were considered statistically significant when the *p*-value was <0.05. as indicated as: ns *p* ≥ 0.05 (not significant), * *p* < 0.05, ** *p* < 0.01, *** *p* < 0.001, **** *p* < 0.0001.

## 3. Results

### 3.1. ACATs Interact with Caveolins in Caveolae

The amino acid sequence spanning residues 80–101 on the N-terminal side of the membrane-insertion region of caveolin-1 is critical for its interactions with many partner proteins and has been termed the caveolin scaffolding domain [36]. We noted that both ACAT1 and ACAT2 contain several putative caveolin-binding motifs/peptides (Supplementary Table S1), suggesting that ACATs may interact with Cav1 in caveolae.

Lipid rafts/caveolae are enriched in cholesterol and sphingolipids, which form highly ordered lipid structures that can be isolated in vitro either with or without detergents. When detergents are used, lipid rafts are recovered as low-density, detergent-resistant membranes (DRMs) [37,38]. To test whether ACAT1 associates with Cavs in caveolae, we isolated DRMs and detergent-sensitive membranes (DSMs) from human fibroblast extracts using either the strong detergent Triton X-100 or the milder detergent Brij-98, followed by Western blot analysis (Figure S1A). When Brij-98 was used, most of the ACAT1 signal was recovered in the DRM fraction, whereas the cholesterol sensor protein SCAP of the SREBP pathway [39] resided almost exclusively in the DSM fraction. As controls, the lysosomal membrane marker LAMP2 was predominantly detected in the DSM fraction, and a minor portion of the ER marker protein calnexin was also detected in the DSM fraction. In contrast, when Triton X-100 was used, essentially all ACAT1 signals were detected in the DSM fraction.

We next performed similar experiments using extracts from hACAT1 cells, which are mutant CHO cells lacking endogenous ACAT activity but stably expressing human ACAT1 [30]. DRMs and DSMs were prepared using increasing concentrations (2–4%) of the detergent CHAPSO. Western blot analysis (Figure S1B) showed that at high CHAPSO concentrations (4%), human ACAT1 was predominantly recovered in the DRM fraction. In contrast, at both low and high CHAPSO concentrations, SCAP and 3-hydroxy-3-methylglutaryl-CoA reductase (HMGCR), the rate-limiting enzyme in cholesterol biosynthesis [40], were consistently localized to the DSM fraction. Together, these results (Figure S1A,B) suggest that in the presence of detergent, ACAT1 associates with Cavs in lipid raft/caveolae membranes, a compartment that appears to exclude other key components of the cholesterol biosynthesis pathway. Using a similar approach, we had previously demonstrated that the cellular cholesterol efflux protein ABCA1 was predominantly recovered in the DRM fraction in human fibroblast extracts [29], consistent with earlier findings using different methodologies [10].

**Figure 1:**
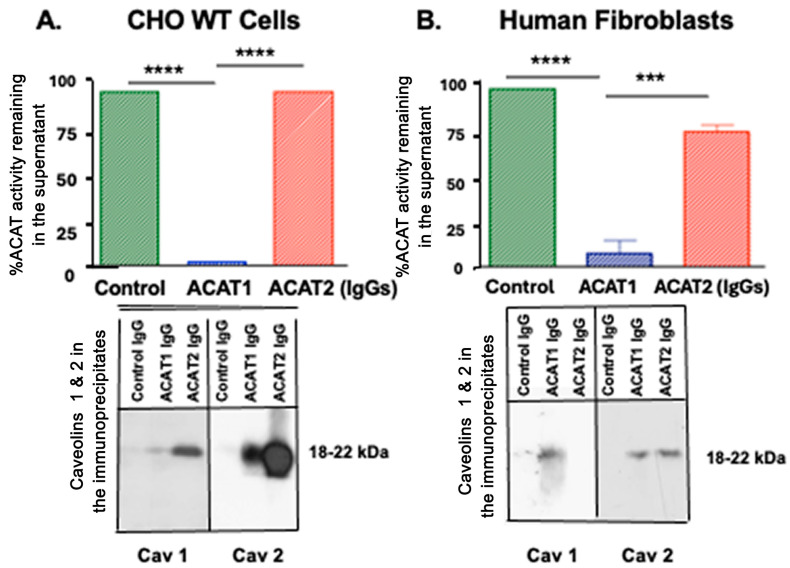
Immunoprecipitation of ACAT1 and ACAT2. Immunoprecipitation analyses of ACAT1 and ACAT2 were performed in either WT CHO cells (**A**) or human fibroblasts (**B**). A total of 1.2 × 10^7^ cells were seeded in 150 cm^2^ dishes. Cells were solubilized in buffer containing 2% CHAPS and 1 M KCl, supplemented with protease inhibitors. Cell extracts were centrifuged at 100,000× *g* for 1 h to obtain solubilized enzyme fractions. For each sample, 0.5 mL of cell lysate (2 mg of protein/mL) was immunodepleted with 5 µg of IgG against ACAT1 (DM10), ACAT2 (DM54), or control rabbit IgG. Depleted lysates were aliquoted (30 µL per assay), and ACAT activities were measured in triplicate as described in Section 2. The top panels show ACAT activity remaining in the supernatants after immunodepletion. The bottom panels show detection of caveolin-1 or caveolin-2 in the immunoprecipitates by Western blotting using antibodies against caveolin-1 or caveolin-2, respectively. Data are expressed as means ± SEM (*N* = 2). Results are from one of two experiments with similar results. *** *p* < 0.001; **** *p* < 0.0001. The original Western blot images are shown in the Supplementary Materials.

To test whether ACAT1 or ACAT2 binds directly to caveolins, we performed immunoprecipitation experiments in detergent, using extracts from CHO cells (Figure 1A) and human fibroblast cell extracts (Figure 1B).

Cell extracts were solubilized with CHAPS in the presence of high levels of salt (1 M KCl), and immunoprecipitation was performed using ACAT1-specific antibodies. Following immunoprecipitation, both supernatants and pellets were analyzed either for ACAT enzymatic activity or by Western blotting (see Section 2). In both cell types examined, the results show that ACAT1 strongly interacts with caveolin-1 and caveolin-2. Similar experiments were performed using ACAT2-specific antibodies. The results show that the specific anti-ACAT2 antibodies did not bring down detectable ACAT2 (as monitored by Western blot and by ACAT enzyme activity measurements).

The ACAT2-specific antibodies did bring down sufficient Cavs, which were detectable by Western blotting, while the non-specific antibodies failed to bring down any ACATs or Cavs. We interpret these results as being due to the low abundance of ACAT2 present in CHO cells and human fibroblasts (Hf cells). ACAT2 in these cells is present at low levels and is not readily detectable by Western blot or enzyme activity assays; however, ACAT2-specific antibodies can co-immunoprecipitate sufficient ACAT2 and associated Cavs for detection by Western blot.

The results in Figure 1 indicate that both ACAT1 and ACAT2 interact with Cavs. These results also demonstrate that ACAT1 and ACAT2 do not interact with each other to form heteromeric complexes. In subsequent studies described in this manuscript, we focus on studying ACAT1.

**Figure 2:**
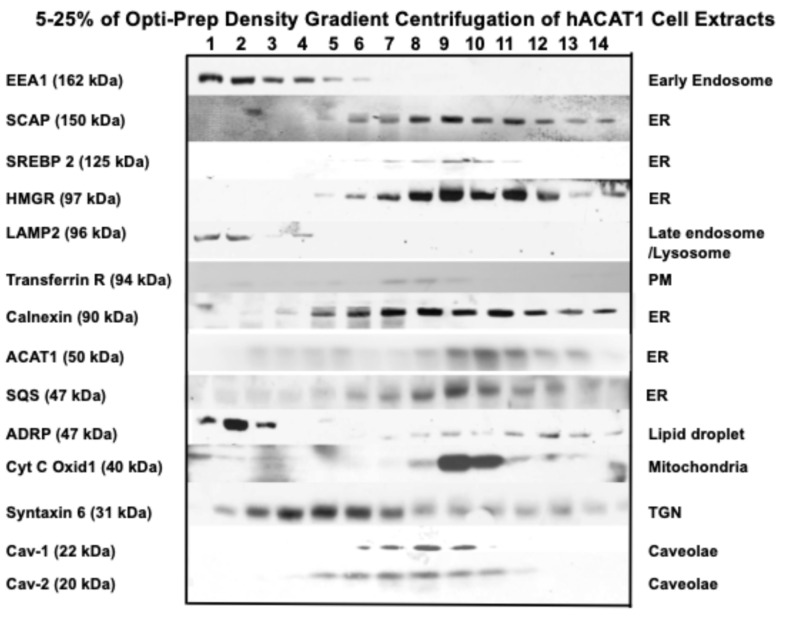
Subcellular distribution of ACAT1, caveolins, and various organelle marker proteins in His-ACAT1 cell extracts. OptiPrep density gradient centrifugation experiments were conducted as described in the Methods. The trans-Golgi network (TGN) was enriched in fractions 3–7. *N* = 2. Results are from one of two experiments with similar result. The original Western blot images are shown in the Supplementary Materials.

To minimize the possibility that the interaction observed between ACATs and Cavs was an artifact of detergent solubilization, we developed a detergent-free approach. Whole-cell extracts of hACAT1-expressing cells were subjected to density gradient centrifugation to isolate ER-enriched membrane fractions, which were then subjected to immunoadsorption using ACAT1-specific antibodies to isolate ACAT1-containing membrane vesicles. Density gradient centrifugation fractionated the extracts into 14 equal fractions and partially separated various membrane organelles (Figure 2).

We used transferrin receptor (transferrin R) as the PM marker, which showed that the PM is enriched in fractions 7–9. This result confirms previous work by Urano et al. [33], who used biotinylated proteins as the PM marker. Fractions 3–8 contain caveolins. Additional caveolins, particularly caveolin-2, were also detected in fractions 9–11.

ACAT1 was broadly detected in ER membrane fractions (fractions 5–11) and in fractions containing mitochondrial markers (fractions 8–11). Fractions 9–11 were enriched in classical ER marker proteins, including calnexin, squalene synthase (SQS), HMGCR, sterol regulatory element-binding protein-2 (SREBP-2), and SREBP cleavage-activating protein (SCAP), and they also contained mitochondrial membranes. Fractions 9–11, which were relatively high in buoyant density, were pooled and subjected to immunoadsorption analysis.

**Figure 3:**
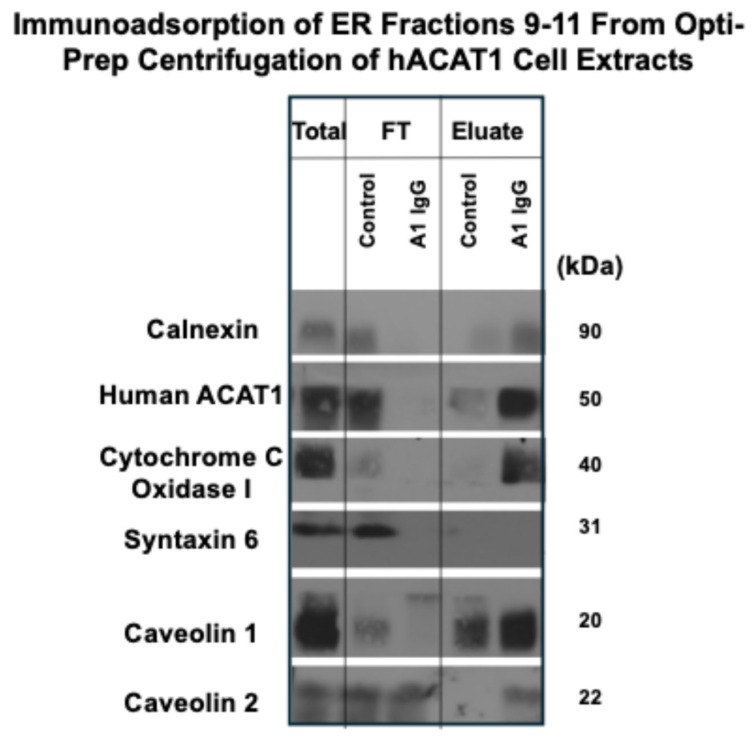
Immunoadsorption analysis of fractions 9–11 enriched in ACAT1, caveolins, and mitochondria in His-ACAT1 cell extracts. Fractions 9–11 were collected from the experiment described in Figure 2 and used for immunoadsorption experiments. Immunoadsorption was conducted using A1 IgG or control IgG as described in the Methods. Control IgG refers to non-specific rabbit IgG, whereas A1 IgG refers to anti-ACAT1 IgG. FT indicates flow-through, and the eluate refers to proteins eluted from antibody-bound microbeads with SDS-PAGE sample buffer. *N* = 2. Results are from one of two experiments with similar results. The original Western blot images are shown in the Supplementary Materials.

Analysis of the immunoadsorbed material (Figure 3) showed that the relatively dense ACAT1-containing membrane vesicles were enriched in caveolin-1 and caveolin-2 but contained minimal amounts of syntaxin-6, a TGN marker. These membranes were also enriched in cytochrome c oxidase subunit IV (COX IV). These results show that fractions 9–11 are enriched in ER–mitochondria membrane contact sites, also known as mitochondria-associated membranes (MAMs). Previous studies have demonstrated that ordered lipid domains form at ER–mitochondria contact sites [41], and ACAT1 has previously been identified as a component of MAMs [42,43,44,45].

**Figure 4:**
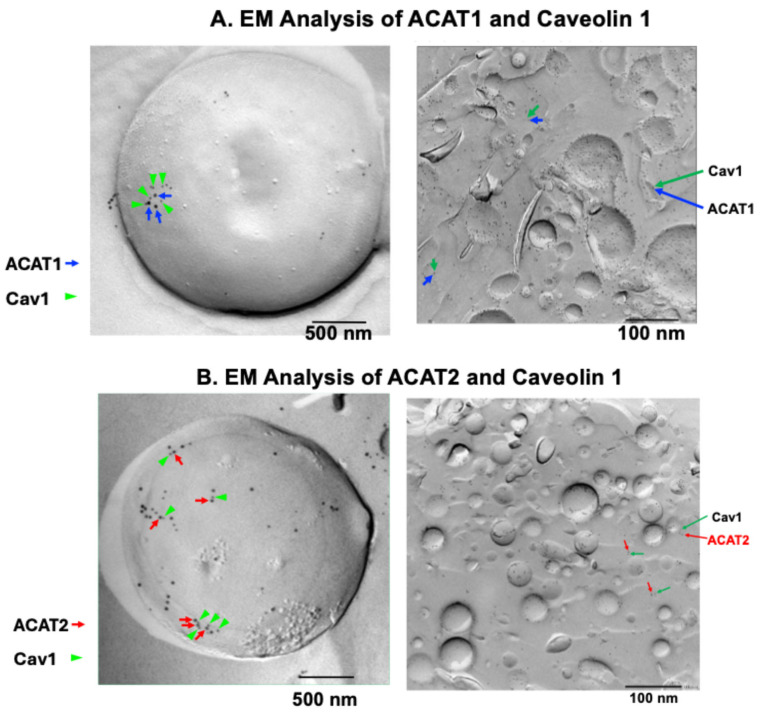
Immunogold electron microscopy of His-tagged ACAT1 or His-tagged ACAT2. Subcellular fractionation was performed on cells expressing (**A**) His-ACAT1 or (**B**) His-ACAT2. Extracts from 1 × 10^8^ cells were subjected to OptiPrep density gradient ultracentrifugation, and fractions 9–11 (described in Figure 2 and Figure 3) were collected. These fractions were further centrifuged at 100,000× *g* for 45 min at 4 °C to isolate ER membrane fractions. The resulting pellet was fixed with 1% paraformaldehyde for 15 min, diluted with PBS to a final concentration of 0.1% paraformaldehyde, centrifuged again, and resuspended in 50 µL of PBS. The pellets were frozen, and freeze-fracture replicas were prepared. The samples were processed for immunogold labeling as described in [34] using anti-His IgG and anti-Cav1 IgG. ACATs were labeled as large, dark gold particles, whereas Cav1 was labeled as small, light gold particles, *N* = 2.

To test the possibility that ACAT1 and Cavs are in close proximity, fractions 9–11 were used to perform EM analyses. The results (Figure 4A,B, left: high resolution; right: low resolution) show that in these membranes, only a small portion of ACAT1 (represented as large, dark gold particles) and a small portion of Cav1 (represented as small, light gold particles) colocalize (i.e., are in close proximity), while the rest of these two molecules are not close to each other. The same observation applied to ACAT2. These data suggest that a subset of ACAT1 and/or ACAT2 molecules may associate with caveolin-1 in these membranes.

A low-magnification image (shown on the right) contains multiple membrane vesicles—approximately 20–50 vesicles are visible (each round or oblong object, whether concave or convex, represents a single vesicle, while the flat portions represent ice). The low-magnification images were taken randomly. Since the original fixed ER-enriched fraction contained a mixture of ER, MAM, and other membrane vesicles, the images reflect that mixture accordingly. Without specific immunolabeling markers, the origin of individual vesicles cannot be identified.

Many of the gold particles indicated by arrows or arrowheads in the low-magnification images appear to be located within the flat ice regions. This observation suggests that some of these particles may represent nonspecific antibody binding rather than specific labeling. Moreover, the size of the gold particles is difficult to determine in the low-magnification images. Therefore, the high-magnification images shown on the left were used for detailed analysis.

The vesicles observed in the high-magnification images are mostly in the range of 50–200 nm in diameter, which is generally considered characteristic of microsomal vesicles. For both A and B, high-magnification images are shown on the left, whereas low-magnification images are shown on the right.

The results of Figure 4A,B show that only a small fraction of ACAT1 signals co-localize with the caveolin-1 signals in certain membrane vesicles. Since OptiPrep cannot provide clean separation among various membrane fractions, it is possible that the membranes shown in Figure 4 actually represent the plasma membrane. We consider this possibility unlikely based on previous studies: immunofluorescent confocal microscopy studies in intact cells showed that the ACAT1 signal is highly enriched in the ER, with some signal overlapping with that of mitochondria [46]; this result was corroborated by other studies using different technologies, including biochemical cross-linking and cell fractionation, demonstrating that certain portions of the ACAT1 signal are present in the MAM [43,44,45]. We therefore interpret the membrane vesicle that exhibits colocalization between ACAT1 or ACAT2 with caveolin-1 (Figure 4) as being derived from the MAM. In specialized cells such as macrophages, microscopic analysis showed that some ACAT1 signal can be detected near the TGN [47]. Importantly, no ACAT1-positive signal has been reported at the plasma membrane.

The results in Figure 1 demonstrate that, in detergent solution, ACAT1 forms apparent associations with Cav1 and Cav2. In contrast, the result of the electron microscopy analysis (Figure 4) shows that, in membranes enriched in the ER and mitochondria, only a small portion of ACAT1 and Cav1 colocalize; the rest of these two molecules are not close to each other. To explain the discrepancy, we suspect that the apparent “tight interaction between ACAT and Cav” observed in Figure 1 might be influenced by the presence of the detergent CHAPS. It is possible that Cav1 and ACAT1 have an intrinsic affinity for each other, but they are spatially separated in membranes. The detergent may disrupt the membrane and remove the barrier between these two molecules, allowing Cav1 and ACAT1 to encounter each other and become tightly bound in solution. Alternative explanations cannot be excluded.

**Figure 5:**
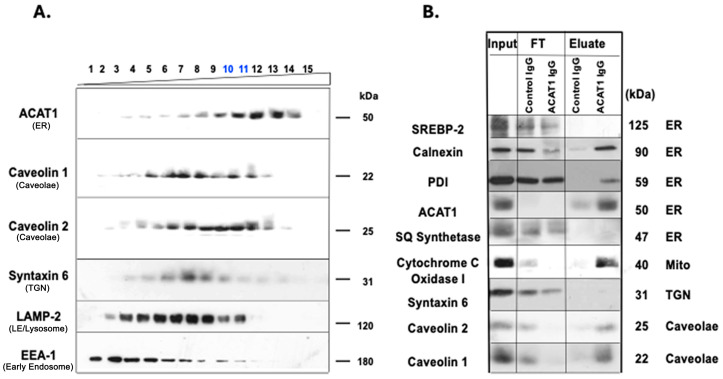
OptiPrep density gradient fractionation and immunoadsorption analysis of ACAT1-associated proteins in HeLa cell extracts. Experiments were conducted essentially as described in Figure 2 and Figure 3 but using HeLa cell extracts. (**A**) OptiPrep density gradient fractionation. One 150 cm^2^ dish of HeLa cells maintained in 10% FBS was lysed using a stainless-steel homogenizer, and 1 mL of post-nuclear supernatant (PNS) was fractionated on a 5–25% OptiPrep density gradient (40,000 rpm for 3 h in an SW41 rotor at 4 °C). Equal aliquots from each fraction were analyzed by SDS-PAGE and immunoblotting using antibodies against the indicated subcellular marker proteins. (**B**) Immunoadsorption analysis. Fraction 10 obtained from the density gradient fractionation shown in Figure 5A was used as the starting material. Abbreviations used are the same as those used in Figure 3. *N* = 1. The original Western blot images are shown in the Supplementary Materials.

The experiments described in Figure 3 used CHO cells expressing human ACAT1 by transfection. To validate this finding in a human cell line, we next examined cells that endogenously express human ACAT1. HeLa cell extracts were subjected to density gradient centrifugation (Figure 5A), and an ER-rich fraction (fraction 10; relatively high in buoyant density) was isolated and subjected to immunoadsorption analysis (Figure 5B).

These results (Figure 5) were consistent with those obtained in CHO cells, implying that some ACAT1 and some caveolin-1 are located in the same membrane, namely, the mitochondria-associated ER membrane (MAM), or in other membranes tightly associated with the MAM.

### 3.2. Caveolin-1 Deficiency Increases Re-Esterification of LDL-Derived Cholesterol from the Lysosome

Because portions of Cavs may form association with portions of ACAT1 (Figure 4A), we hypothesize that Cavs may regulate the re-esterification of cholesterol derived from LDL-cholesteryl linoleate. To test this hypothesis, mouse embryonic fibroblasts (MEFs) were prepared from C57BL/6 mice that were wild-type (WT), Cav1 knockout (KO), or ACAT1 KO, and the trafficking of [^3^H]-labeled LDL-derived cholesteryl linoleate was monitored using a previously described assay [32,35].

**Figure 6:**
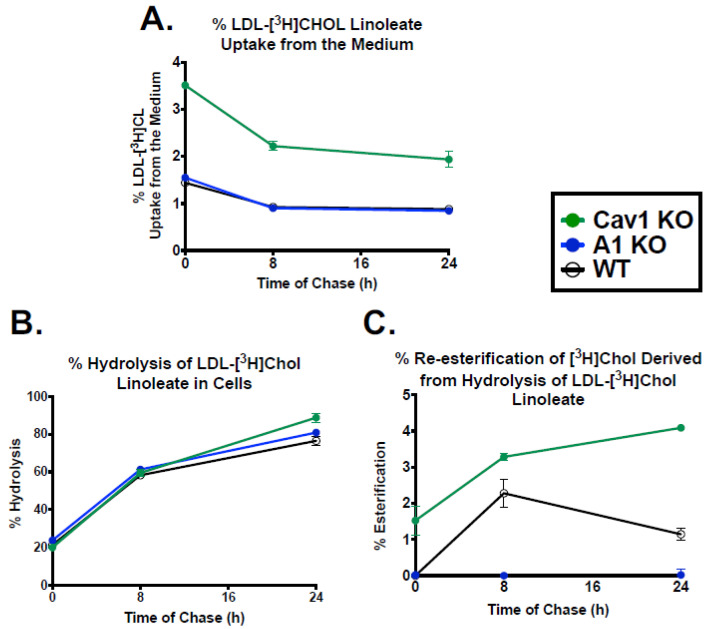
(**A**) %[^3^H]-cholesteryl linoleate (CL)-LDL uptake; (**B**) % hydrolysis of [^3^H] CL-LDL; and (**C**) % re-esterification of [^3^H]-cholesterol derived from [^3^H]-CL-LDL in mouse embryonic fibroblasts (MEFs). The experiments were conducted at 37 °C in a CO_2_ incubator. MEFs were seeded at a density of 0.25 x 10^6^ cells per well in 6-well plates and cultured in 10% FBS for 24 h. After rinsing twice with PBS, cells were incubated in DMEM containing 5% delipidated serum (DeS) and 35 µM oleic acid. The medium was refreshed the night before the pulse–chase experiment. Cells were pulsed with [^3^H]-CL-LDL (31 µg protein and ~47,200 cpm per dish) in DMEM containing 10% DeS and 35 µM oleic acid for 3 h, washed once with DMEM containing 0.1% fatty acid-free bovine serum albumin (BSA), washed twice with PBS, and chased for 0, 8, or 24 h in DMEM containing 0.1% BSA. At each time point, cells were harvested and subjected to lipid extraction and analysis [35] for measurements of hydrolysis and re-esterification. “%[^3^H]-CL-LDL uptake” is defined as the amount of [^3^H]-CL-LDL remaining in the cell extracts divided by the total counts of [^3^H]-CL-LDL added during the pulse. % hydrolysis was calculated as the sum of [^3^H]-cholesterol and [^3^H]-cholesteryl oleate divided by the amount of [^3^H]-cholesteryl linoleate taken up by the cells. Re-esterification was calculated from the amount of [^3^H]-cholesteryl oleate formed divided by the sum of [^3^H]-cholesterol and [^3^H]-cholesteryl oleate formed. *N* = 2. Results are from one of two experiments with similar results.

The major cholesteryl ester carried in LDL is cholesteryl linoleate (CL). Goldstein and colleagues [25] prepared [^3^H]cholesteryl linoleate, fed it to human fibroblasts, and demonstrated that this material enters the lysosomal compartment and undergoes acid lipase–dependent hydrolysis; [^3^H]CL in the lysosomes is converted to [^3^H]cholesterol and linoleate. Under oleate-feeding conditions, a portion of the free [^3^H]cholesterol liberated from lysosomes is re-esterified by ACAT (using oleoyl coenzyme A to produce [^3^H]cholesteryl oleate) in a time-dependent manner. [^3^H]cholesteryl oleate can be separated from [^3^H]cholesteryl linoleate. We adopted the procedure of Goldstein et al. [25] and measured the counts in [^3^H]cholesteryl oleate divided by the sum of counts in [^3^H]cholesterol and [^3^H]cholesteryl oleate, expressed as % re-esterification, to monitor ACAT activity in intact cells. We also incorporated a pulse–chase step [32], as described in the Figure 6 legend.

The results showed that, at zero time after the pulse, the amount of uptake of LDL-[^3^H]CL (defined in the Figure 6 legend) in Cav1 KO cells was much higher than that in WT and ACAT1 KO cells (Figure 6A; 3.5% in Cav1 KO vs. 1.4% in WT and 1.6% in ACAT1 KO). During the chase period of 8 h or 24 h, the residual [^3^H]CL value decreased more rapidly in Cav1 KO cells but still remained significantly higher than that in WT and ACAT1 KO cells.

We measured the % hydrolysis of LDL-CL at zero time; these values were comparable among the three cell types (WT: 21%, Cav1 KO: 20%, ACAT1 KO: 24%). The % hydrolysis increased over time during the chase and remained comparable across all three cell types (Figure 6B). We also measured % re-esterification (Figure 6C) and found that in Cav1 KO cells, the % re-esterification at 0 h, 8 h, and 24 h after the chase was significantly higher than that in WT cells. As controls, the % re-esterification in ACAT1 KO cells remained essentially negligible.

We compared the % hydrolysis and % re-esterification values in MEF cells versus CHO cells under the same pulse–chase conditions. Both the % hydrolysis and re-esterification efficiencies in WT MEFs were relatively lower than the values reported in CHO cells [33]. Because CHO cells exhibit rapid growth, we speculate that the culture conditions for MEF cells may require optimization for LDL trafficking assays.

The results in Figure 6C support previous results by Lisanti and colleagues [9], who reported a significant increase in cholesteryl ester content in Cav1-deficient MEFs. Mechanistically, we cannot rule out the possibility that the increased re-esterification of [^3^H]cholesterol derived from [^3^H]CL (Figure 6C) is partly due to the increased LDL-CL uptake observed in Cav1 KO cells (Figure 6A).

### 3.3. A Working Model

**Figure 7:**
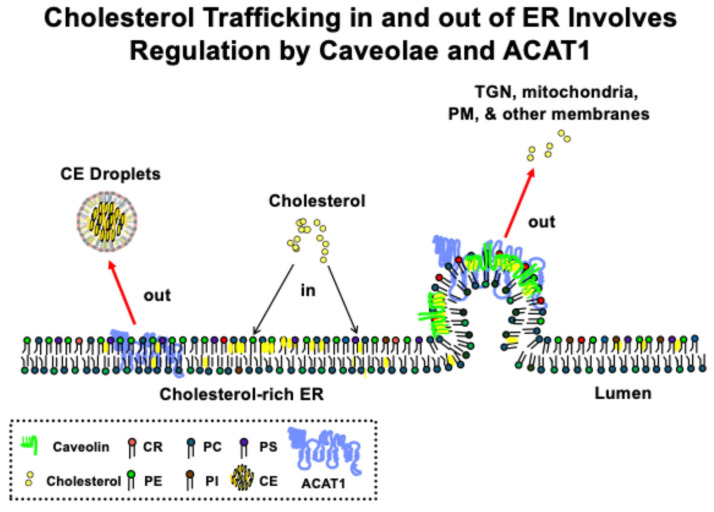
Working model illustrating cholesterol trafficking in and out of the ER involving ACAT1 and caveolae. Abbreviations: CE, cholesteryl esters; PC, phosphatidylcholine; PI, phosphatidylinositol; PS, phosphatidylserine; CR, ceramides. The diagram emphasizes the abundance of cholesterol and ceramide (CR) in caveolae within the ER membrane, versus ER membranes that lack caveolae. The relative distribution of caveolin and lipids in the two membrane leaflets was randomly drawn because these parameters are not yet well-defined in the literature. Black arrows represent NPC-dependent and NPC-independent cholesterol transport pathways delivering cholesterol to the ER membrane. The red arrow on the left represents the action of ACAT1 (blue), which resides in the ER and converts cholesterol to CEs for storage in lipid droplets. The red arrow on the right represents the action of caveolins (green), which transfer cholesterol from caveolae-like microdomains in the MAM to other membranes, including the TGN, mitochondria, and the PM, thereby facilitating cholesterol recycling, eventually leading to cholesterol disposal to the cell exterior. At the MAM, a portion of caveolins in caveolae binds to a portion of ACAT1 and restricts ACAT1’s access to cholesterol. Overall, Cav1 and ACAT1 act in concert to promote cholesterol utilization, recycling, and disposal rather than cholesterol storage.

Based on the findings described above, we propose a working model to explain how Cavs regulate cholesterol esterification and trafficking (Figure 7).

Most cholesterol molecules exiting the endo/lysosomal compartment depend on the cholesterol transporters Niemann–Pick type C1 and C2 [48,49] to reach the ER membrane. ACAT1 is broadly distributed in the ER and converts a portion of incoming cholesterol to cholesteryl esters for storage in lipid droplets. ACAT1 is particularly enriched at the MAM, where a subset of ER membranes is closely associated with mitochondria.

A fraction of cholesterol arriving at the ER is sequestered within ER-associated caveolae through binding to Cavs. In the MAM, by interacting with ACAT1 and ACAT2, Cavs restrict access of these enzymes to cholesterol in the ER, thereby limiting esterification. Cavs in the MAM also facilitate cholesterol transfer to adjacent membrane compartments, including the TGN, plasma membrane, and mitochondria, promoting cholesterol recycling and efflux [49,50]. Cholesterol efflux relies on ABCA1, caveolae at the cell surface, and extracellular high-density lipoproteins [51,52], and thus represents an early step in reverse cholesterol transport.

Cholesterol released from the endo/lysosomal compartment may traffic directly to caveolae at the TGN or other membrane compartments without first passing through the ER [33]. In this scenario, Cavs would still function as indirect inhibitors that attenuate ACAT-mediated cholesterol esterification. In addition, Cavs or their associated lipids, such as ceramides in the ER caveolae [22] may also act as direct inhibitors of ACAT enzymes [53]. These possibilities will require further experimental testing.

## 4. Discussion

Caveolins and caveolae play important roles in cellular cholesterol homeostasis; however, their functions are far from fully understood. Caveolins are known to interact with the major cholesterol efflux protein ABCA1, but whether they also directly interact with other key proteins involved in cholesterol metabolism has not been clearly demonstrated previously. In this study, we show by electron microscopy (Figure 4A,B) that a subset of caveolin-1 is in close proximity to a fraction of ACAT1 or ACAT2, suggesting a possible interaction between caveolin-1 and ACAT1, as well as ACAT2. Furthermore, we demonstrate that the loss of caveolin-1 results in increased ACAT1 activity in intact cells. Together, these findings suggest that caveolins may act to attenuate cholesteryl ester formation, perhaps by restricting the availability of cholesterol as a substrate for ACAT1.

Based on these results, we propose a working model in which caveolins regulate cholesterol metabolism within a specialized subdomain of the endoplasmic reticulum, namely the mitochondria-associated membrane (MAM). Previous studies have shown that caveolins localize to ER–mitochondria contact sites and that these regions are enriched in ordered lipid domains. Our data suggest that a portion of ACAT1 is also enriched at the MAM, where it forms apparent tight complexes with a subfraction of caveolins. It is possible that, by sequestering cholesterol within ER-associated caveolae, caveolins limit cholesterol esterification and promote cholesterol trafficking to other cellular membranes.

The relevance of caveolins to cholesterol metabolism in the central nervous system (CNS) further underscores the potential physiological significance of our findings. Cholesterol is an essential lipid in the brain, where caveolins are expressed in astrocytes, specific neuronal populations, and activated microglia [54]. Caveolins are generally considered neuroprotective, and genetic loss of caveolin-1 in mice accelerates neurodegeneration and aging [55]. Notably, the cellular distribution of ACAT1/SOAT1 in the brain closely parallels that of caveolins, with expression in multiple CNS cell types [56]. Functional studies have shown that genetic deletion or pharmacological inhibition of ACAT1 attenuates Niemann–Pick type C1 (NPC1) disease, a severe neurodegenerative disorder [31]. These observations support the idea that limiting cholesterol esterification is neuroprotective. The model proposed in Figure 7 provides a plausible mechanistic framework linking caveolin function to neuroprotection. By restricting cholesterol esterification, caveolins maintain a pool of free cholesterol that is available for membrane trafficking and recycling among intracellular organelles. This mechanism may be particularly important in post-mitotic cells such as neurons, where efficient cholesterol redistribution is critical for membrane maintenance and synaptic function. Future studies will be required to further elucidate how caveolin–ACAT interactions are regulated under physiological and pathological conditions, and how these interactions contribute to neurodegenerative disease processes.

## 5. Conclusions and Limitations of the Current Study

It is well established that cholesterol influx via LDL or other cholesterol-rich sources, such as myelin debris, supplies exogenous cholesterol and increases ACAT1 activity. Conversely, inhibition of ACAT1 enhances cellular cholesterol efflux to the medium. Together, these findings support a central role for ACAT1 in maintaining the balance between cellular cholesterol influx and efflux.

In addition, previous studies have demonstrated that a fraction of ACAT1 localizes to endoplasmic reticulum (ER)–mitochondria contact sites, also known as mitochondria-associated membranes (MAMs), which serve as key hubs for lipid metabolism. With respect to caveolins, multiple reports have implicated caveolins in both cholesterol influx and efflux, and a subset of caveolin-1 (Cav1) has likewise been localized to the MAM. Despite these observations, direct interactions between ACAT proteins and caveolins have not been previously reported.

In this study, based on electron microscopy analysis of ER-enriched fractions (Figure 4A,B), we propose that a subset of ACAT and caveolins co-localize and may interact at the MAM. Functionally, we further demonstrate that loss of Cav1 enhances LDL-mediated cholesteryl ester formation (Figure 6). To integrate these findings, we present a working model (Figure 7) and suggest a new function for ACAT1: ACAT1 acts together with Cavs to promote cholesterol utilization, recycling, and disposal in addition to promoting cholesterol storage.

Several limitations should be noted. First, the evidence for co-localization and potential interaction between ACAT and caveolins is indirect and primarily based on ultrastructural analysis. Second, the functional data are limited. While suggestive, they do not establish a direct mechanistic link between Cav1 and ACAT activity. We have not tested whether the cholesterol status of the cells may modulate the interaction between ACAT1/ACAT2 and caveolins. Third, the proposed model remains preliminary and requires validation through complementary approaches. These include high-resolution live-cell fluorescence microscopy, cross-linking analyses of ACATs and caveolins in intact cells, and biochemical reconstitution experiments to test direct interactions between purified ACAT1 and caveolins in vitro.

Overall, this study provides an initial framework for understanding a potential connection between ACAT1 and caveolins at the MAM, while highlighting the need for further investigation to substantiate and refine the proposed model.

## Figures and Tables

**Table 1:** Potential “Caveolin-Scaffolding Domains” on ACAT1 and ACAT2.

Target	Source	Identifier
ACAT1	Chang laboratory	DM10
ACAT2	Chang laboratory	DM54
ABCA1	Novus Biologicals	NB-400-105
ADRP	Thermo Fisher Scientific	15294-1-AP
β-Tubulin	GenScript	A01717
Calnexin	GenScript	A01234
Caveolin-1	Santa Cruz Biotechnology	SC-894
Caveolin-2	BD Biosciences	610684
Cytochrome c Oxidase I	Santa Cruz Biotechnology	SC-58347
EEA1	Cell Signaling Technology	2411
HMGCR	Invitrogen	MA5-35242
LAMP1	Cell Signaling Technology	D401S
LAMP2	Santa Cruz Biotechnology	SC-18822
PDI	Cell Signaling Technology	2446
SCAP	Invitrogen	MA5-37768
SREBP-2	R&D Systems	AF7119
Squalene Synthase	Santa Cruz Biotechnology	SC-365101
Syntaxin-6	Andrew Peden laboratory	—
Transferrin R	R&D Systems	MAB5746
Vinculin	Millipore	05-386

## Data Availability

The original contributions presented in this study are included in the article/Supplementary Materials. Further inquiries can be directed to the corresponding author.

## Supplementary Materials

The following supporting information can be downloaded at: https://www.mdpi.com/article/10.3390/biom16060838/s1. Table S1. Potential “Caveolin-Scaffolding Domains” on ACAT1/ACAT2. Figure S1A is for Human Fibroblast and Figure S1B is for His-ACAT1-expressing CHO cells. Original Western Blot image for Figures.
