# Supplementary material for: A Subset of Caveolin-1 Interacts with a Fraction of Acyl-CoA:Cholesterol Acyltransferase 1 (ACAT1/SOAT1) at an Endoplasmic Reticulum Subdomain to Attenuate Cholesteryl Ester Biosynthesis
Source: Biomolecules. 2026 Jun 8;16(6):838. doi: 10.3390/biom16060838 (PMC13296461; doi:10.3390/biom16060838)
Supplement: Supplementary file 1 [file biomolecules-16-00838-s001.zip › biomolecules-4346573-Original Western blots.pdf]

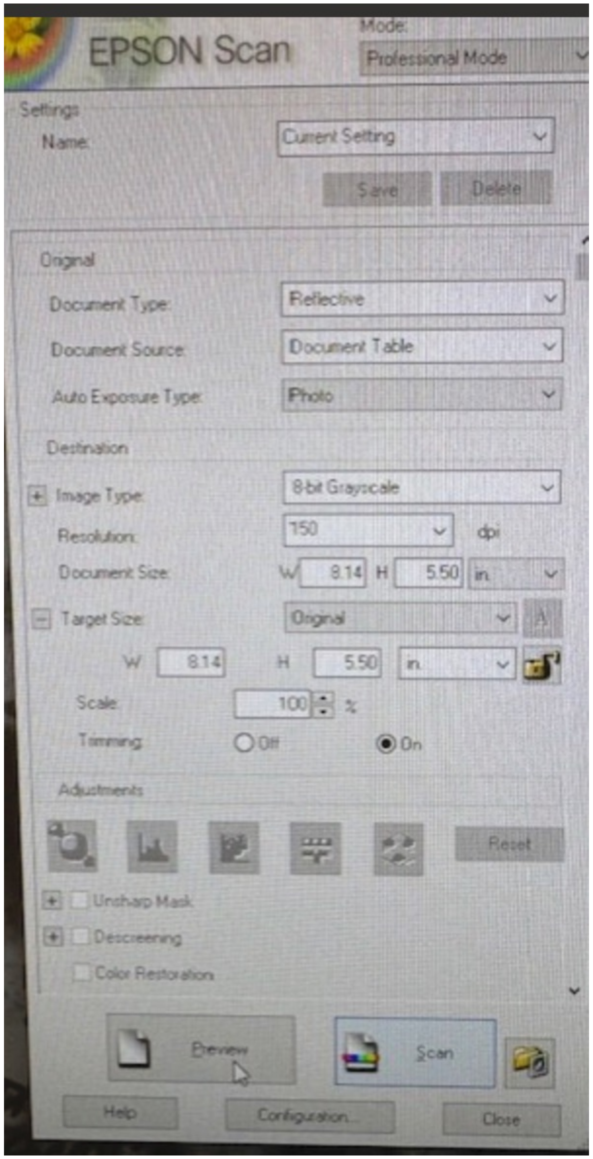

All Western blots were scanned using Epson Scan,
Image Type: 8-bit Grayscale
Resolution: 150 dpi

Submitted figure
Figure 1

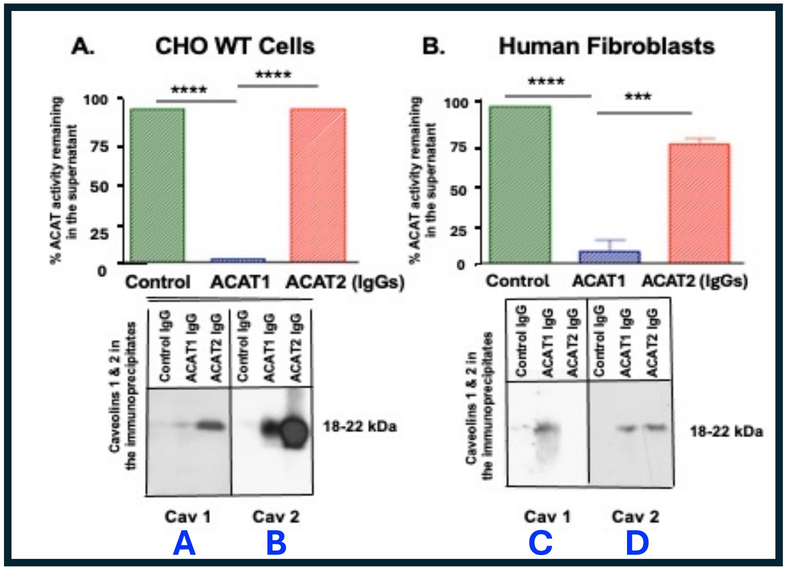

Original blots

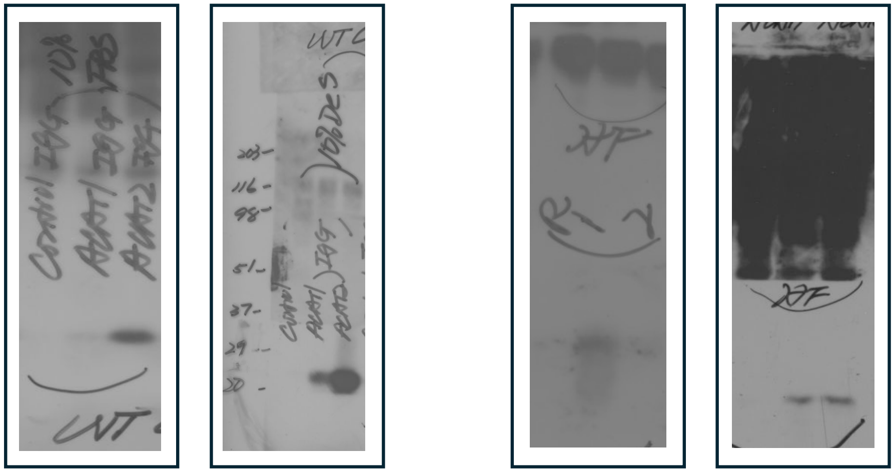

A B C D

Figure 2 Opti Prep Density Gradient Centrifugation (Part 1)

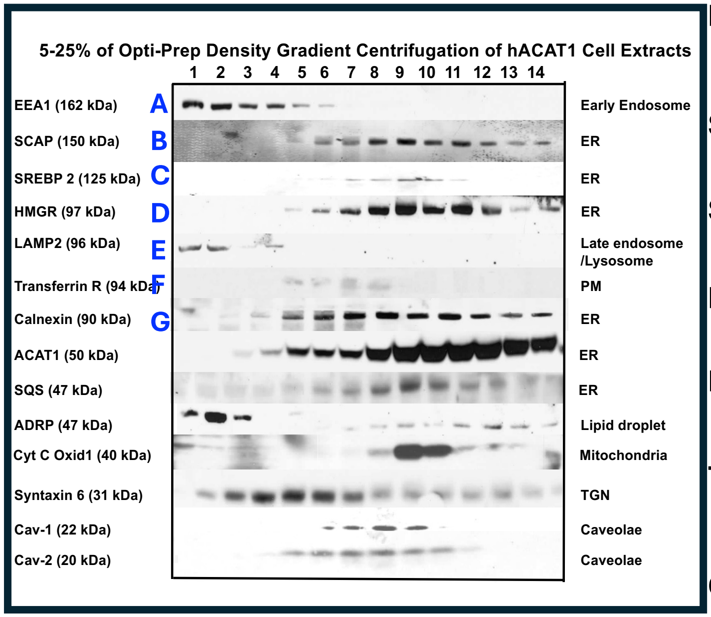

Submitted figure
Figure 2, (Part 1)

EEA 1 (162 kDa)

SCAP (150 kDa)

SREBP2 (125 kDa)

HMGR (97 kDa)

LAMP2 (96 kDa)

Transferrin R (94 kDa)

Calnexin (90 kDa)

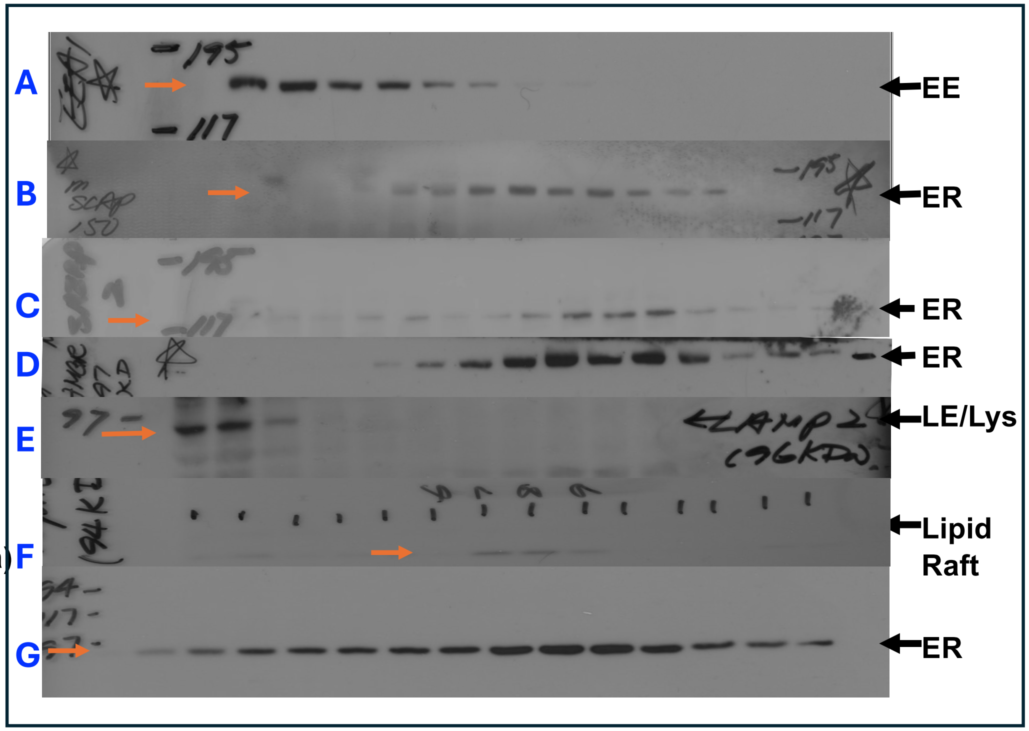

Original blots

Figure 2 Opti Prep Density Gradient Centrifugation (part 2)

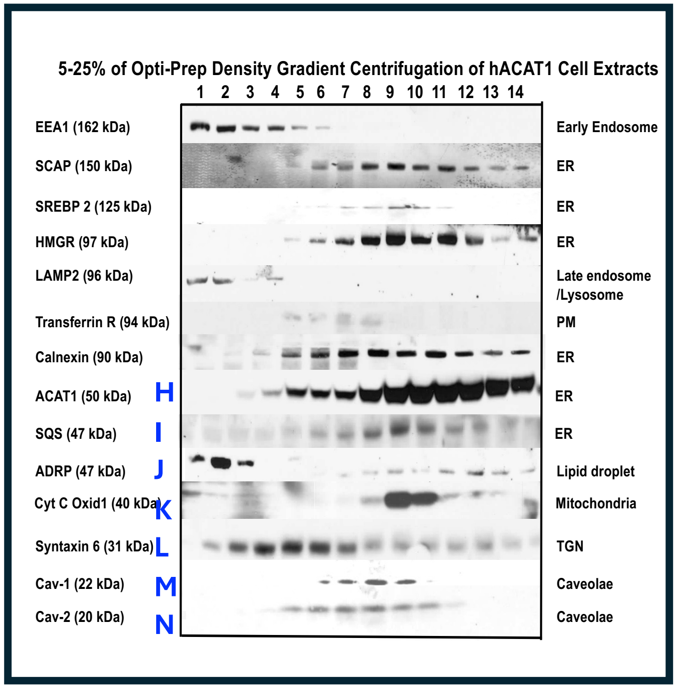

Submitted figure
Figure 2, part 2

ACAT1 (50 kDa)

SQS (47 kDa)

ADRP (47 kDa)

Cyt C Oxidase

Syntaxin6 (31 kDa)

Cav 1 (22 kDa)

Cav 2 (20 kDa)

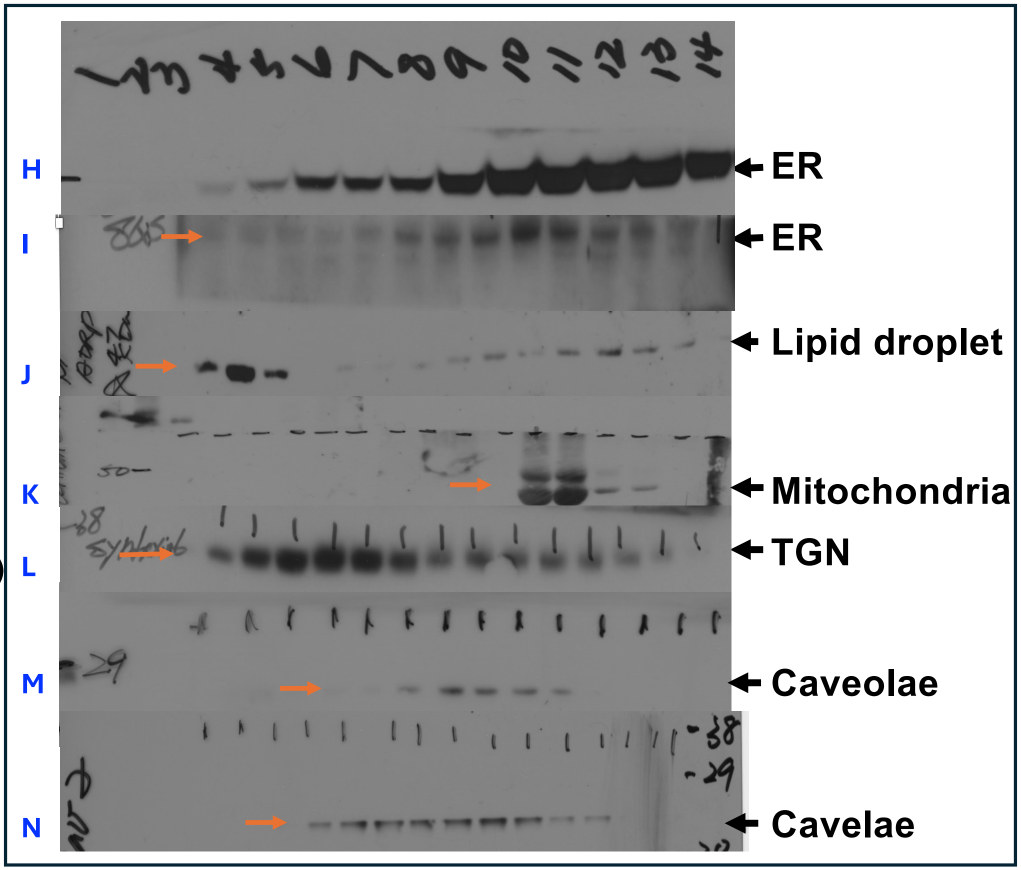

Original blots

Figure 3. Immunoadsorption of ER fractions 9-11 from OptiPrep centrifugation of hACAT1 cell extracts

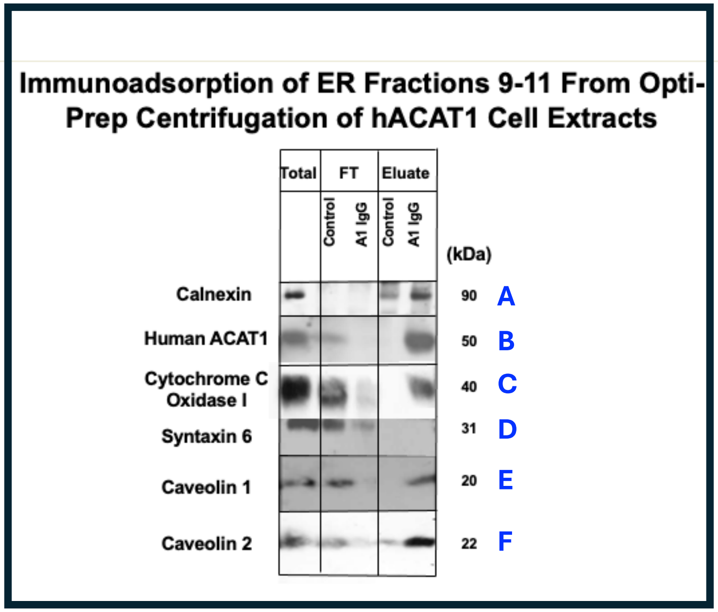

Submitted figure
Figure 3

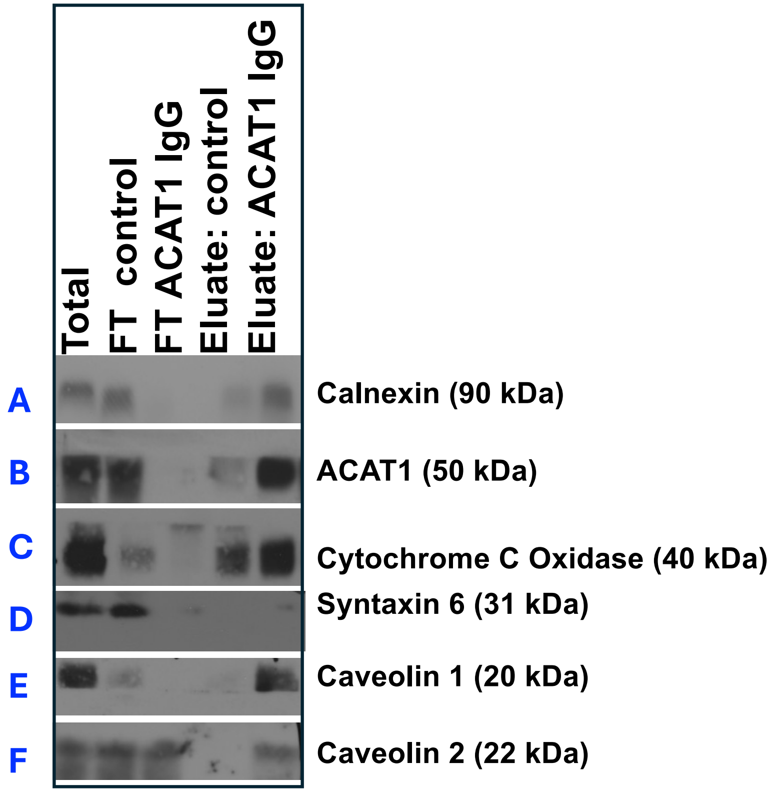

Original blots

Figure 5A

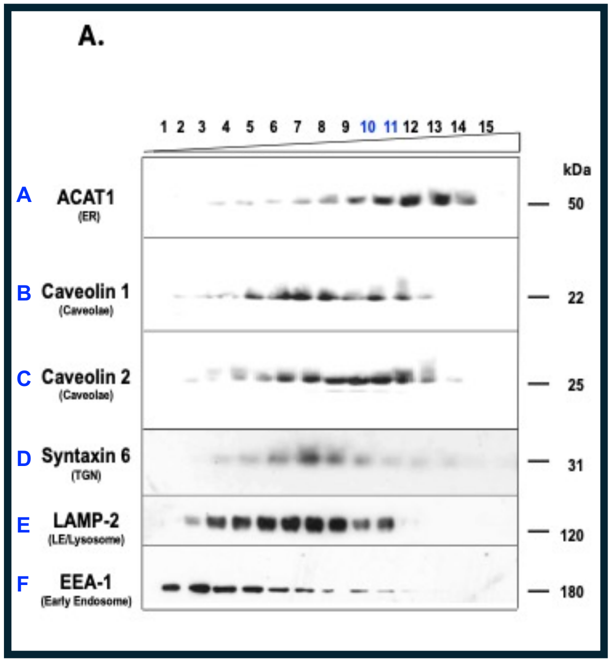

Submitted figure
Figure 5A

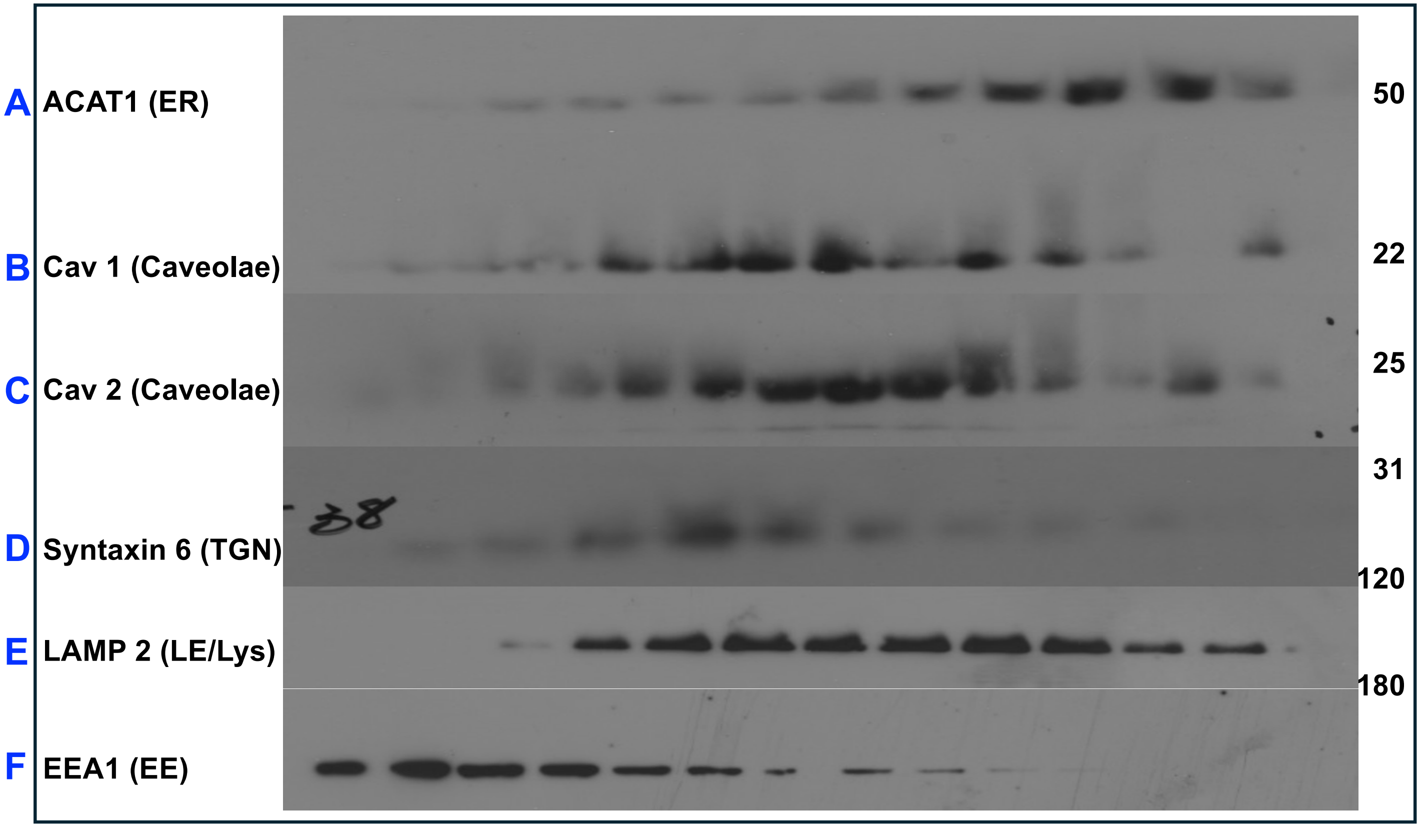

Original blots

Figure 5B: Immunoadsorption Analysis

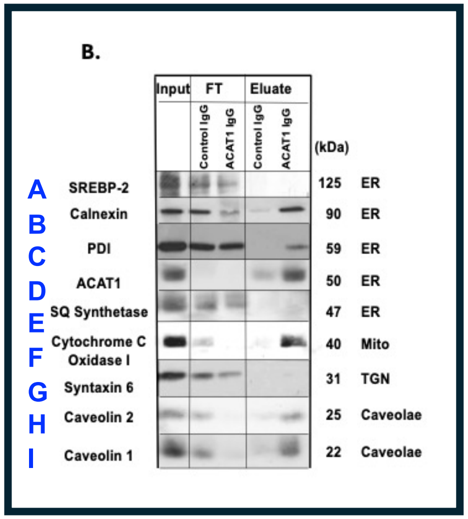

Submitted figure
Figure 5B

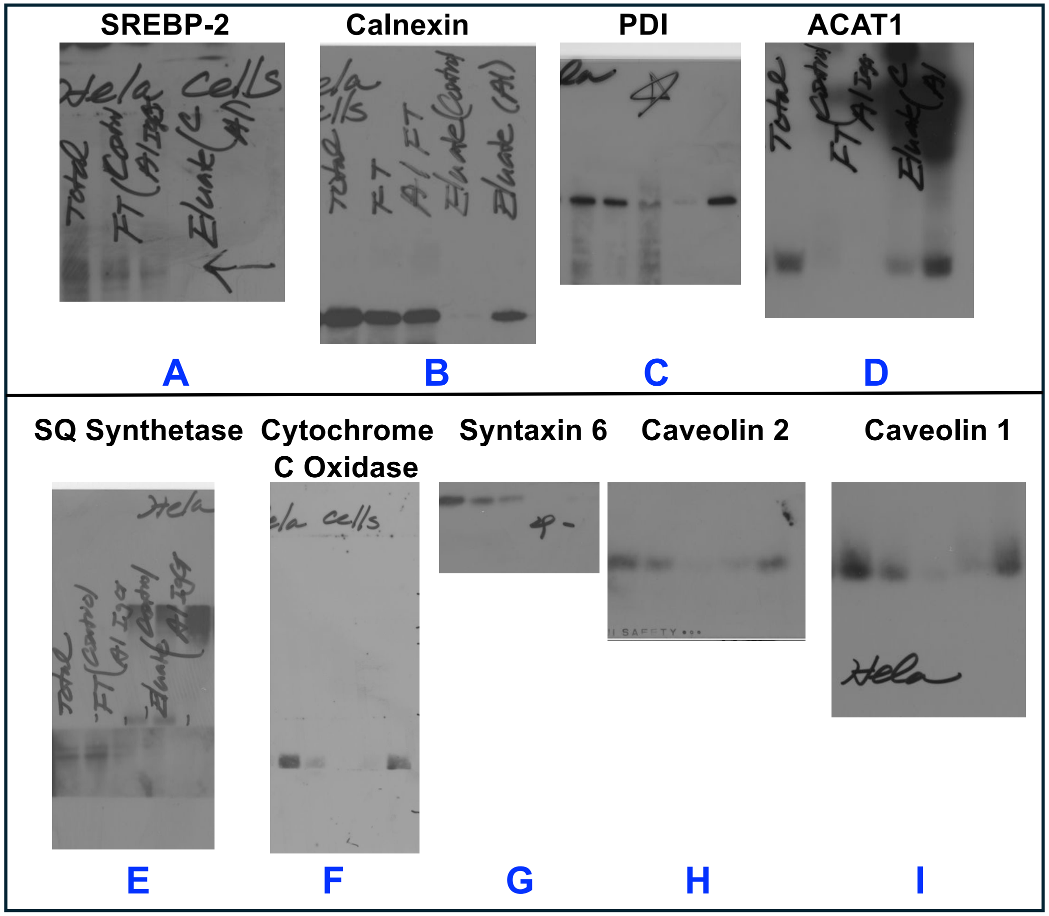

Original blots

Supplementary Figure S1A

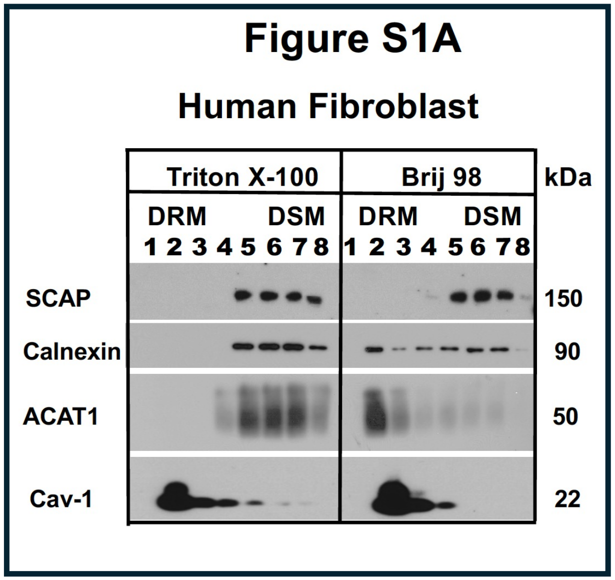

Submitted figure
Supplementary Figure S1A

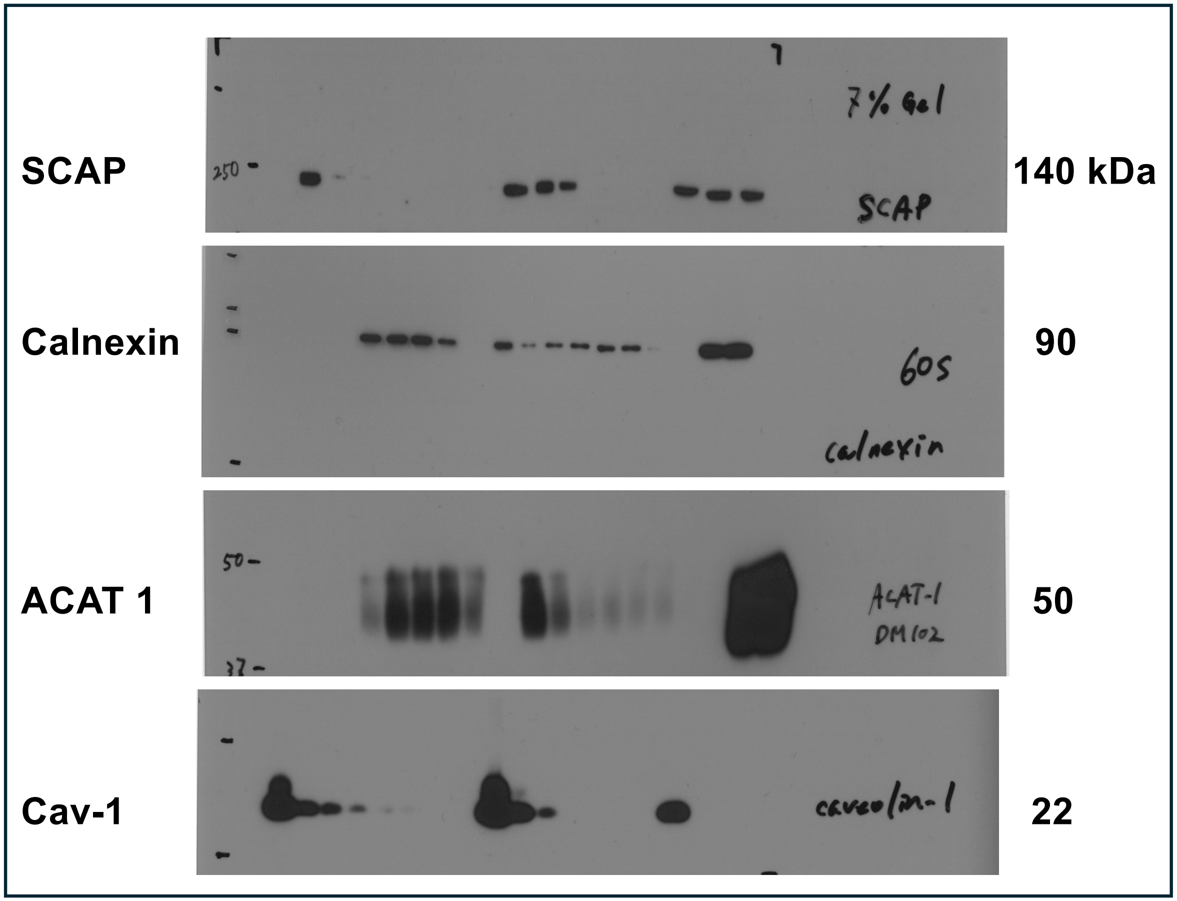

Original blots

Supplementary Figure S1B

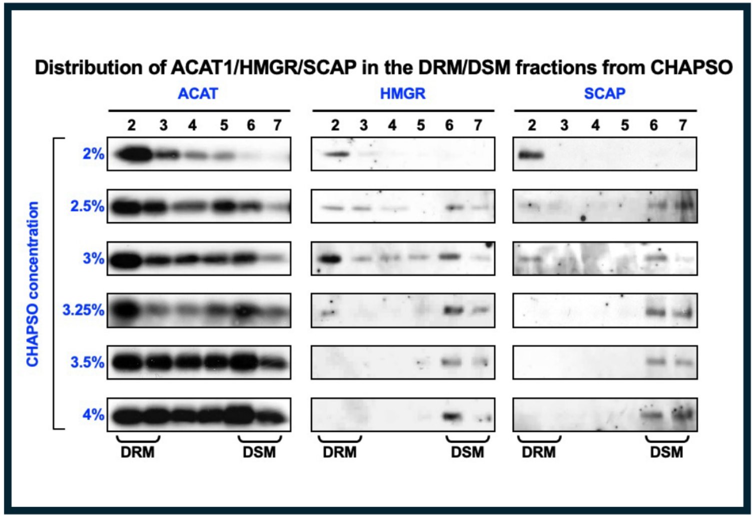

Submitted figure
Supplementary Figure S1B

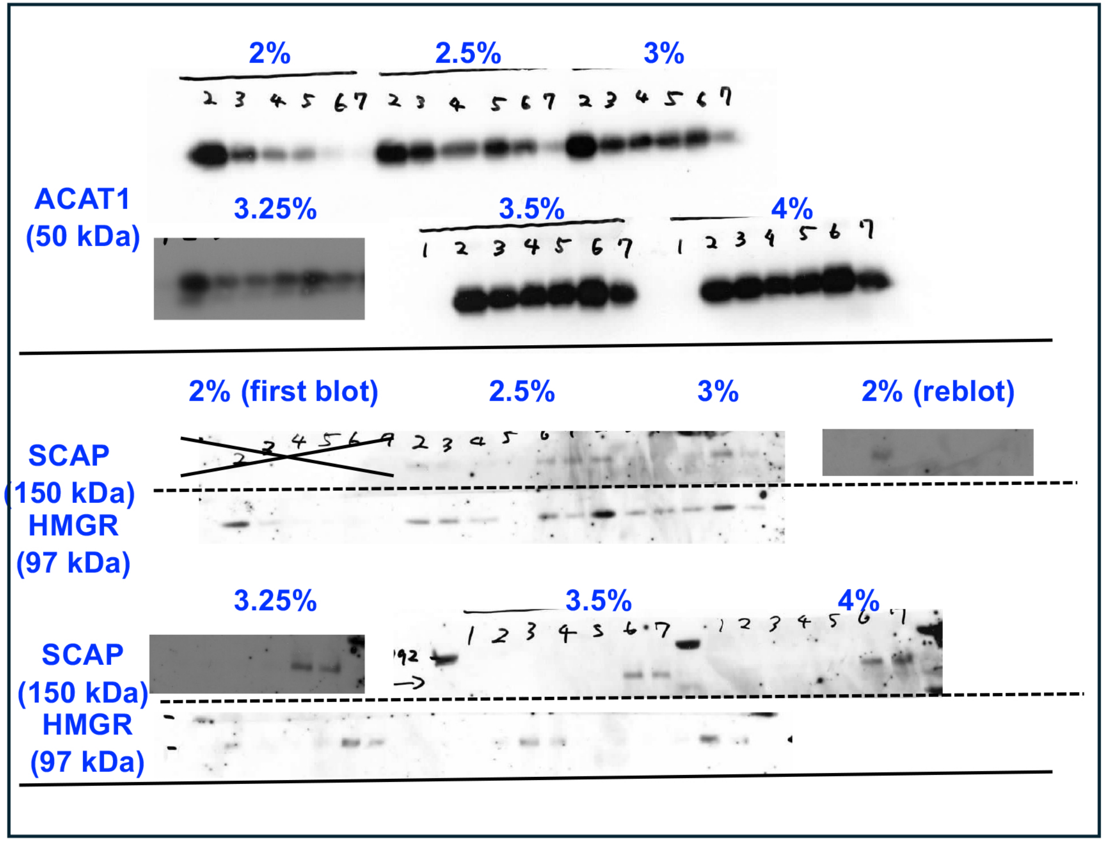

Original blots
