# Supplementary material for: A Subset of Caveolin-1 Interacts with a Fraction of Acyl-CoA:Cholesterol Acyltransferase 1 (ACAT1/SOAT1) at an Endoplasmic Reticulum Subdomain to Attenuate Cholesteryl Ester Biosynthesis
Source: Biomolecules. 2026 Jun 8;16(6):838. doi: 10.3390/biom16060838 (PMC13296461; doi:10.3390/biom16060838)

Table S1. Potential "Caveolin-Scaffolding Domains" on ACAT1/ACAT2

ACAT1 (2nd TMD)<br>181 186 189<br>F xxxx W xx W<br><br>ACAT2<br>163 168 171<br>L xxxx V xx V	ACAT1 (3rd C.L.)<br>320 322 327<br>W x Y x x x x F<br><br>ACAT2 (C.L.)<br>294 296 301<br>W x Y xxxx F	ACAT1 (5th TMD)<br>331 336 339<br>F xxxx Y xx Y<br><br>ACAT2<br>305 310 313<br>L xxxx Y xx F	ACAT1 (4th C.L.)<br>408 413 416<br>W xxxx Y xx Y<br><br>ACAT2 (C.L.)<br>382 387 390<br>W xxxx F xx Y	ACAT1 (4th C.L.)<br>431 433 438<br>Y x Y xxxx W<br><br>ACAT2<br>405 407 412<br>Y x Y xxxx R/W	ACAT1 (8th TMD)<br>471 476 479<br>F xxxx F xx F<br><br>ACAT2<br>444 449 452<br>F xxxx L xx F
ACAT1 (8th TMD)<br>479 481 486<br>F x F xxxx F<br><br>ACAT2<br>453 455 460<br>F x V xxxx L	ACAT2 (C.L.)<br>268 273 276<br>F xxxx Y xx F<br><br>ACAT1<br>294 299 302<br>V xxxx Y xx F	ACAT2 (C.L.)<br>286 291 294<br>Y xxxx Y xx W<br><br>ACAT1<br>312 317 320<br>Y xxxx T xx W	ACAT2 (membrane)<br>438 440 445<br>F x F xxxx F<br><br>ACAT1<br>464 466 471<br>L x V xxxx F		

(C.L.: Cytosolic Loop; TMD: Transmembrane domains)

Figure S1A

Human Fibroblast

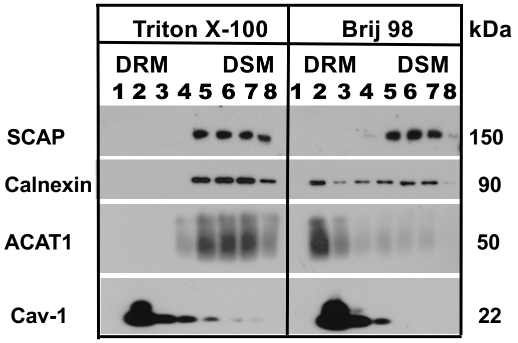

Figure S1B

His-ACAT1–expressing CHO cells

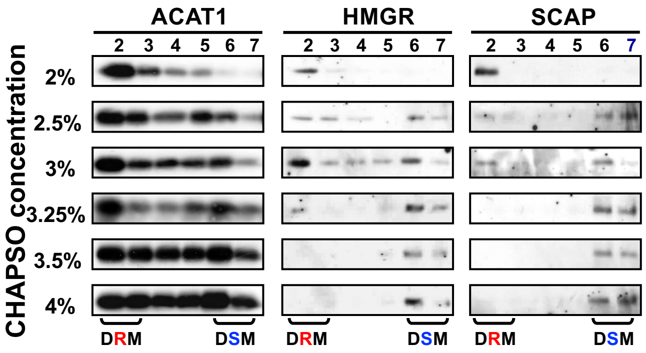

Supplement: Supplementary file 1 [file biomolecules-16-00838-s001.zip › biomolecules-4346573-supplementary.pdf]
